# Discovering overlapped protein complexes from weighted PPI networks by removing inter-module hubs

**DOI:** 10.1038/s41598-017-03268-w

**Published:** 2017-06-12

**Authors:** A. M. A. Maddi, Ch. Eslahchi

**Affiliations:** 1 Department of Electrical and computer Engineering, Isfahan University of Technology, Isfahan, 1983963113 Iran; 2 Department of Computer Sciences, Faculty of Mathematics, Shahid Beheshti University, Tehran, 1983963113 Iran; 3 School of Biological Sciences, Institute for Research in Fundamental Sciences (IPM), Tehran, 193955746 Iran

## Abstract

Detecting known protein complexes and predicting undiscovered protein complexes from protein-protein interaction (PPI) networks help us to understand principles of cell organization and its functions. Nevertheless, the discovery of protein complexes based on experiment still needs to be explored. Therefore, computational methods are useful approaches to overcome the experimental limitations. Nevertheless, extraction of protein complexes from PPI network is often nontrivial. Two major constraints are large amount of noise and ignorance of occurrence time of different interactions in PPI network. In this paper, an efficient algorithm, Inter Module Hub Removal Clustering (IMHRC), is developed based on inter-module hub removal in the weighted PPI network which can detect overlapped complexes. By removing some of the inter-module hubs and module hubs, IMHRC eliminates high amount of noise in dataset and implicitly considers different occurrence time of the PPI in network. The performance of the IMHRC was evaluated on several benchmark datasets and results were compared with some of the state-of-the-art models. The protein complexes discovered with the IMHRC method show significantly better agreement with the real complexes than other current methods. Our algorithm provides an accurate and scalable method for detecting and predicting protein complexes from PPI networks.

## Introduction

Many biological functions, in living organisms are accomplished by proteins. In fact, proteins are the smallest operating units in cells whose cell organization and functions comprehension depends on their behavior. A protein rarely operates stand-aloan. In other words, proteins operate in groups which are called complex^1^. A complex is made up of proteins that are all physically connected at the same time. It is notable that a protein complex should not be mistaken by a functional module. A functional module is defined by high density of interactions within a group of proteins, where a group is said to have high density when the amount of intergroup interactions are more than intragroup ones. An approach to study these crucial molecules is recognition of different complexes. We know that, a single cell of a simple organism consists of thousands of proteins, so there are millions of potential complexes related to them. Although available accurate experimental methods can determine the authenticity of proposed complexes, the mentioned experimental processes are not possible and reasonable, due to the extreme number of these candidate complexes^2^. It seems that computational approaches can be a suitable alternative for detecting these complexes^3^. Extracting protein complexes from protein interaction networks is one of these computational approaches. In recent decades, many powerful experimental methods have been proposed to extract a large amount of protein-protein interactions (PPIs)^4^. Tandem Affinity Purification with mass spectrometry (TAP-MS)^5^, Yeast-Two-Hybrid (Y2H)^6^, Co-immunoprecipitation (Co-IP) and Protein-Fragment Complementation Assay (PCA)^7^ are examples of these high-throughput techniques. This collection of PPIs is usually known as PPI network. PPI networks can be modeled as an undirected graph, where nodes denote proteins and edges represent interactions between these proteins. In such networks, complexes are considered as dense subgraphs because it is reasonable to assume that the number of inner interactions between members of one complex is usually more than the number of outer interactions of its members^8, 9^. By making such an assumption, the problem of detecting protein complexes changes to the traditional graph clustering problem.

We know that the existence of several limitation in experimental methods causes considerable noise (false positive and false negative) in the production process of PPI networks^8^. Although, there is not any certain solution for reducing the noise, some suggestions have been made by some methods. For example, Chua *et al*.^10^ and Brun *et al*.^11^ have proposed two algorithms which use FS-weights^12^ and CD-distance respectively. In addition, some of proteins are multifunctional and simultaneously serve in more than one complex. Therefore complexes may have overlap^13^. It is worth mentioning that not all of the protein-protein interactions have the same reliability and time of occurrence^14^. The reliability of an interaction is often shown by the weight of corresponding edge in PPI networks^15^ while the time is often ignored. Clustering methods should be capable of handling noise, weights and occurrence times of interactions in PPI network.

Due to the variety of available complex detection algorithms, there is no categorization that covers all types of complex detection methods. The primary idea and also type of information which are used in the algorithms, are usually the main axis to classify them. Hence, all available complex detection algorithms can be divided into two main categories. The first category consists of all algorithms that don’t use any biological information except PPI networks. The second category covers the algorithms that use various kinds of biological information in addition to PPI networks. This information is used for making better decisions. In both categories, all algorithms are different types of graph clustering algorithms. The algorithms in the first category are classified into five main groups^16, 17^. (1) Local neighborhood Density search (LD); in which every cluster is initialized with a single node or a group of nodes which construct a dense subgraph. In every step of the algorithm, one or more nodes can be joined to a cluster or to be discarded. The possibility of proposing overlapped clusters is one of the advantages of methods which use this strategy. For example, MCODE^18^ and ClusterONE^13^ belong to this category. (2) Cost-based Local search (CL); the methods in this category decompose the graph into some parts in every step. This graph decomposition is led by a cost function for accessing to a better partitioning. Such methods are often unable to produce overlapped clusters, which is an significant disadvantage of methods in this category^19^. The importance of producing overlapped clusters is due to the large amount of proteins that belong to several protein complexes simultaneously^20^. The core of RNSC algorithm (without the filtering step) is a famous example of such methods^21^. (3) Flow Simulation (FS); the main idea of the methods of this category is the behavior of a fluid in canals and spread of information on a network. The MCL^4^ and RRW^22^ methods are the best examples for this category. They use random walk theorem for implementing their approach. (4) Clique finding methods (CF); algorithms in this category, predict clusters by merging, mixing or deleting the different types of cliques or k-cores. CMC^8^ and CFinder^19^ are samples of this category. (5) Other traditional graph clustering methods; there are often a few methods which don’t have any prominent idea. These methods are unique in their characteristics. Therefore, these algorithms are put in a separate category. AP is an example of this category^23^. Its idea is the same as the popular k-center clustering algorithm which is implemented on weighted graph instead of multi-dimensional vector space^23^. It is also possible to classify algorithms in the second category based on considerable diversity of the types of biological information which is used by them^24^. We only focused on the first category of these algorithms for two main reasons. First, our knowledge and technology is not sufficient for extracting all biological information so, our perspective of biological rules is limited and incomplete. Hence, the significant existing noise and information defects may cause deviation and bias in the extracted biological information and the algorithm’s results. Second, an improvement on the algorithms of the first category can improve the results of algorithms in the second category automatically.

Complex detection methods which are using PPI networks, have a limited accuracy. The large amount of noise (false positive and false negative interactions) are responsible for this fault. Previously, biologists generally had concurred that the amount of connections between vertices in a PPI network are closely related to the their biological importance, hence hubs were more likely to be lethal genes^25^, whereas later it was found that this correlation might not be completely true^26^. On the other hand, Han *et al*. have proposed a binary hub classification which divided hubs into two groups, ‘party hubs’ and ‘date hubs’^27^. Date hub refers to a group of vertices that have many connections with other vertices but in different times. This group of vertices emerges in the form of hubs, when we have a static snapshot of all the occurred interactions, as PPI networks. While party hubs are high degree vertices which appear as global connectors in the PPI networks^28^. Similarly Liu *et al*. classified hubs into two types, ‘module hubs’ and ‘inter-module hubs’^14^. Based on this classification the hubs in a module are recognized as module hubs and the hubs which connect modules to each other are considered as inter-module hubs. Comparing these two classifications, it seems that inter-module hubs are date hubs and module hubs are party hubs. As a result, module hubs are important biological hubs in which their presence is crucial in clusters, while inter-module hubs are unessential or even fake hubs and if necessary, they can be ignored. The more in-depth analysis has been provided by Batada *et al*.^28, 29^.

Thus, probably by eliminating inter-module hubs not only do we have a better-separated network with less noise^28^, but also we consider different occurrence time for protein interactions indirectly. Considering the hubs has recently received much attention. For example Liu *et al*. and Yong *et al*. have considered the biological properties of hubs and have tried to detect protein complexes by removing all hubs in network^30, 31^. Since these methods are classified as the second category, so we were not able to compare their methods with the methods in the first category.

Here we propose a new protein complex detection method from PPI networks which is classified as the first category ‘LD’. The main idea of this method has been based on eliminating noise in networks via removing hubs. In this approach, some of the hubs were removed at the beginning stage. This group of hubs included both module hubs and inter-module hubs. In fact, our study show that many high degree hubs are inter-module hubs in the PPI networks which are denser while these hubs change to module hubs in the case of sparser networks. Then a greedy growth process were used for creating primary clusters from different single nodes. After that, some of the eliminated hubs were added to the primary clusters based on the density of PPI network and modularity concept. This concept helped us to add module hubs to appropriate primary clusters and filter the inter-module hubs. Final clusters were presented by merging highly overlapped primary clusters and filtering the sparse clusters. The experimental results demonstrate that our algorithm (IMHRC) outperforms other protein complex detection methods, especially ClusterONE algorithm, that is a state-of-the-art method^32^.

## Results

Before presenting the results of our study, we have discussed datasets, evaluation metrics and Gold Standards which were used to assess the results of complex detection algorithms. Then the results of the methods are presented.

### Evaluation metrics

Comparing the outputs of complex detection algorithms with a predefined gold standard set is one of the common ways to assess their performance. Existing significant amount of overlap between real complexes in the gold standard sets and also between predicted complexes, cause the difficulty in comparison methods. On the other hand, it is possible to match a real complex with more than one predicted complex and vice versa. In addition, the matching between predicted complexes and real complexes is often partial. So we need to use some standard criteria in order to calculate the amount of matching between the gold standard and predicted complexes.

One of the common criteria in literature is the geometric accuracy (Acc) which has been introduced by Brohee and van Helden^33^. It is the geometric mean of clustering-wise sensitivity (Sn) and clustering-wise positive predictive value (PPV). Given *n* complexes in the gold standard as references and *m* predicted complexes, let *t*_*ij*_ denote the number of common proteins between reference complex *i* and predicted complex *j* and also let *N*_*i*_ denote the number of proteins in the reference complex *i*. Sn, PPV, and Acc are defined as followed:

1 $$Sn=\,\frac{{\sum }_{i=1}^{n}\mathop{{\max }}\limits_{j}\{{t}_{ij}\}}{{\sum }_{i=1}^{n}{N}_{i}}$$

2 $$PPV=\,\frac{{\sum }_{j=1}^{m}\mathop{{\max }}\limits_{i}\{{t}_{ij}\}}{{\sum }_{j=1}^{m}{\sum }_{i=1}^{n}{t}_{ij}}$$

3 $$Acc=\,\sqrt{Sn\times PPV}$$

Sn measures the fraction of proteins in the reference complexes that is detected by predicted complexes. Since the availability of a giant component can increase the amount of Sn, PPV was used. In fact, protein aggregation in one predicted complex inflates Sn while putting every protein into to the correct predicted complexes which is the same as reference complexes, can maximize the PPV. So accuracy criterion (Acc) was used for balancing the two measures. It should be noted that using Acc cannot turn them into a perfect criterion for evaluating complex detection algorithms. Assume that there is a perfect complex detection algorithm whose output is the same as reference complex sets. Sn gets the maximum value on this algorithm. But this is not true about the PPV. As a matter of fact, because of the overlapping property, there are some proteins which belong to more than one predicted complex. So the numerator of PPV is always smaller than its denominator. It means that although overlap property is one of the intrinsic properties of complexes, the PPV criterion would not be maximized when overlap exists and this is an obstacle.

Nepusz *et al*. used the Fraction and MMR criterion to overcome this issue^13^. If *P* is denoted the set of predicted clusters and *C* is denoted the set of gold standard complexes, the fraction criterion is defined as following:

4 $${N}_{c}=|\{c|c\in C,\exists \,p\in P,O\,(p,c)\ge \omega \}|$$

5 $$Fraction=\frac{{N}_{c}}{|C|}$$

As mentioned later, $O(p,c)$ which is called as the matching score, calculates the extent of matching between a reference complex *c* and a predicted complex *p*. So these criteria show the fraction of gold standard complexes which are matched by at least one predicted cluster. The threshold *ω* was set to 0.25. By choosing 0.25 for *ω*, it is guaranteed that at least half of the proteins in a matched gold standard cluster is distinguished by at least half of the proteins in a matched predicted cluster. To evaluate MMR, a bipartite weighted graph was constructed which one of its parts associated to the reference complexes and another associated to the predicted complexes. The matching score between every member of one part with each member of another part was calculated by the equation (12) and was considered as a weighted edge in the graph, if its value was greater than 0.2. By running a maximum weighted bipartite graph matching algorithm, we obtained a one-to-one mapping between members of two groups with the maximal match. The value of MMR criterion is equal to the normalized maximal match which is total weight of selected edges, divided by the number of the reference complexes. Nepusz *et al*. have proposed sum of the Accuracy, MMR and Fraction criterions for comparing the performance of the complex detection algorithms^13^. They showed ClusterONE dominates other complex detection methods, and introduced ClusterONE as a state-of-the-art method. In addition, recently Feng *et al*. have introduced ClusterONE as the state-of-the-art complex detection method and have proposed a new supervised learning method that has achieved a better performance than ClusterONE^32^. Since in the learning step of this algorithm biological information are used, we can put this algorithm in the second category. So we compared our experimental results with ClusterONE and other best complex detection methods in the first category.

### Gold Standard set

For evaluating result of the methods, two gold standards were used as benchmarks. These gold standards include the recent version of the MIPS catalog of protein complexes and the Gene Ontology based protein complex annotations from SGD. The MIPS catalog has a hierarchical structure, so the complexes may be composed of several subcomplexes which are available at most in five hierarchy levels deep^13^. We extracted all complexes from all MIPS categories which consist of at least three and at most 100 proteins. It should be mentioned that the MIPS category 550 was excluded, because of all its complexes is predicted by computational methods. Also, we used Saccharomyces Genome Database (SGD) as another source for extracting the second gold standard set. SGD includes Gene Ontology (GO) annotations for all yeast (Saccharomyces cerevisiae) proteins. These GO terms provide biological information which can be used for producing reference complexes. This process has been introduced in refs 13 and 22. Therefore, we used this approach for creating SGD gold standard set which included the reference complexes of at least three and at most 100 protein. In this experiments, the threshold for matching between a predicted complex and a reference complex was considered as 0.25 based on equation (12).

### Datasets

In our assessment four experimental yeast PPI datasets were used which include Gavin^1^, Collins^15^, Krogan Core and Krogan Extended^34^. All these datasets are weighted. Weights express the reliability of each interaction which is a value between zero to one. The weights in the Gavin dataset are Socio-affinity index, which measures affinity between proteins. This criterion calculates how many times pairs of proteins are observed together as preys, or a bait and a prey in the data set and then computes their log-odds^35^. All PPIs in the Gavin data set have socio-affinity index larger than five^1^. All chosen PPIs in the Collins data set were selected based on their purification enrichment score which contains the top 9074 interactions, as suggested in the original paper^15^. In these experiments, we also used two different versions of Krogan dataset. All PPIs in the first version which are referred to as Krogan Core, have weights larger than 0.273, while all PPIs in the second version which are referred to as Krogan Extended, have weights larger than 0.101. Generally, all settings and parameters in every dataset were set based on what the original papers have proposed. Moreover, we decided to eliminate self-interactions and isolated proteins from all datasets. Other properties of these networks are shown in Table 1.

**Table 1:** Details of four PPI Network datasets used in the experiments.

Dataset	Release year	#Proteins	#Interactions	Density
Collins	2007	1622	9074	0.007
Gavin	2006	1855	7669	0.004
Krogan Core	2006	2708	7123	0.002
Krogan Extended	2006	3672	14317	0.002

### Evaluation

To assess the robustness of IMHRC against other complex detection algorithms, we selected seven of the best algorithms in this topic. In this paper, we tried to have a comprehensive comparison of all the state-of-the-art complex detection methods which not only have been introduced in the last decade but also their source codes or binary execution files is accessible. Furthermore, these groups of methods only use topological information and don’t use any biological information except PPI networks. These algorithms include: AP^23^, CFinder^19^, CMC^8^, MCL^4^, ClusterONE^13^, Core of RNSC^21^ and RRW^22^. Parameters of all these methods was set to the values that have recommended by their authors or by Nepusz *et al*. in ref. 13. In fact, Nepusz *et al*. have calculated the best setting for every algorithm on each datasets. In evaluation, the best setting for IMHRC algorithm was used too.

### Determination of β and γ

**Figure 1:**
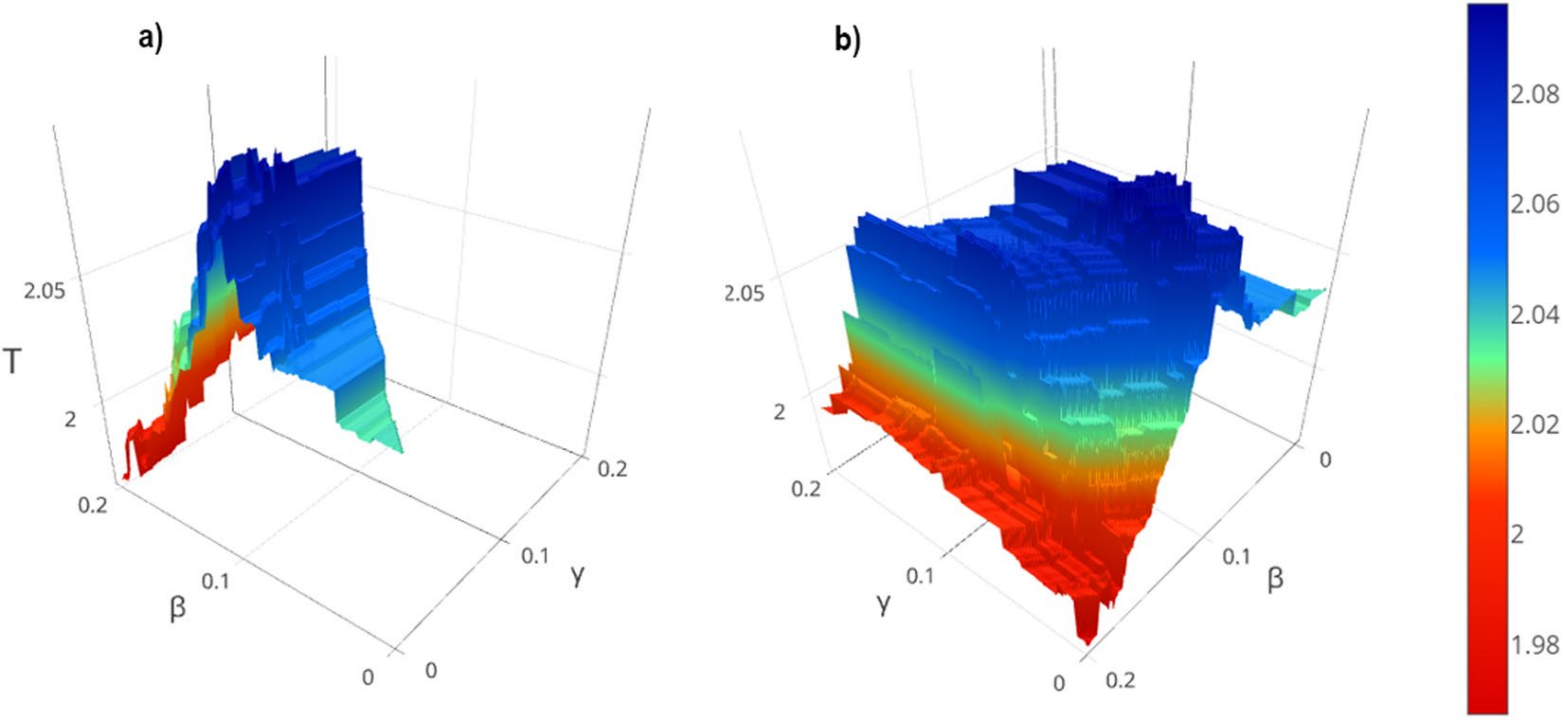
Performance of IMHRC for the different values of β and γ on the Collins dataset and the SGD gold standard. The β and γ axes indicate the number of hubs that have been removed and put back, respectively and T axis specifies the performance of method. (**a**) The back view of surface is shown in this figure. (**b**) The front view of surface is shown in this figure.

**Figure 2:**
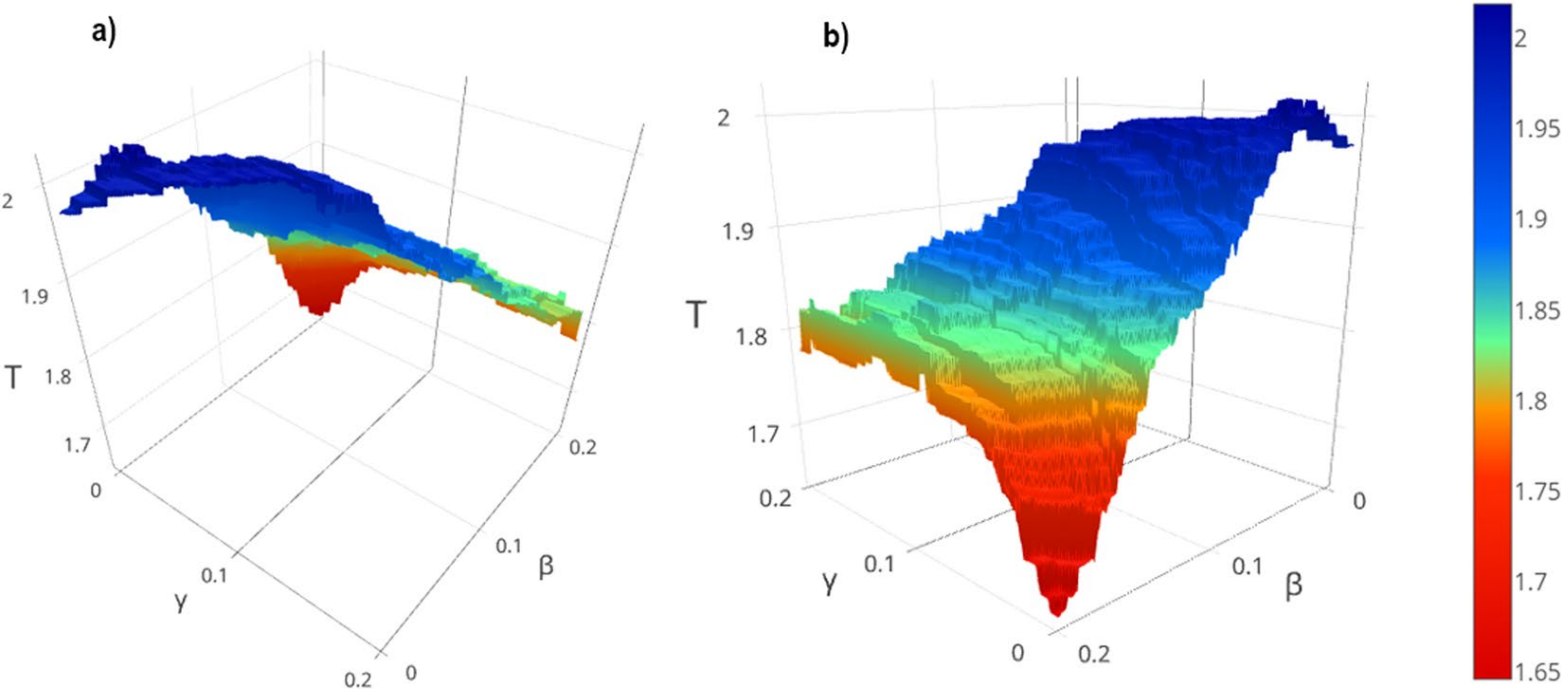
Performance of IMHRC for the different values of β and γ on the Gavin dataset and the SGD gold standard. The β and γ axes indicate the number of hubs that have been removed and put back, respectively and T axis specifies the performance of method. (**a**) The back view of surface is shown in this figure. (**b**) The front view of surface is shown in this figure.

For implementing the idea of removal and putting the hubs back, we used the parameters β and γ. In order to specify the values of β and γ, we calculated the results of IMHRC for each β and γ in the range of $0\le {\rm{\beta }},\gamma \le 0.2$ by considering the change 0.001 of these values in each step. The resulting surfaces are shown in Figs 1 and 2 and Supplementary Figures 1 to 6.

We investigated for the values of β and γ for which performance obtain is the best. Table 2 shows the values of β and γ on the all datasets. The experimental results demonstrate that the best value of β and γ depend to the density of datasets and type of gold standards. It seems that, high values for β and low values for γ are appropriate when the network is dense, while for sparser network, low values should be specified for both of them.

**Table 2:** The threshold of β and γ in IMHRC on all datasets.

Dataset	SGD			MIPS		
	Performance	β	$\gamma$	Performance	β	$\gamma$
Collins	2.096	0.098	0.036	1.689	0.0	0.0
Gavin	2.017	0.042	0.011	1.604	0.01	0.008
Krogan Core	1.832	0.004	0.001	1.456	0.002	0.001
Krogan Extended	1.671	0.015	0.008	1.289	0.001	0.0

### Performance

Table 3 shows the values and settings of all methods. Tables 4 and 5 show the details and the overall performance of the methods based on Accuracy (Acc), Fraction (Frac) and Maximum Matching Ratio (MMR). Variety in the number of real complexes in different datasets is interesting. A real complex was remained in the gold standard set with respect to a dataset, if at least half of its proteins belonged to that dataset. The size of gold standards in Tables 4 and 5 clearly show that krogan datasets are more comprehensive than Collins and Gavin datasets. While the numbers of proteins and interactions in the datasets show that the Gavin and Collins datasets are denser than the other two (Table 1).

**Table 3:** The applied clustering algorithms’ settings in different datasets.

Algorithm	Version	Parameter	SGD				MIPS			
			Collins	Gavin	Krogan Core	Krogan Extended	Collins	Gavin	Krogan Core	Krogan Extended
AP	Unknown(10 Sep 2007)	Preference (P)	0.4	−0.6	0.35	0.3	−0.9	−0.15	0.35	0.4
CFinder		k-clique size(k)	3	4	3	4	3	4	3	3
		Lower link weight threshold(w)	0.0	0.0	0.0	0.0	0.0	0.0	0.0	0.0
	2.0.6	upper link weight threshold(W)	1.0	1.0	1.0	1.0	1.0	1.0	1.0	1.0
		Maximum time of clique searching(t)	0.2	0.2	0.2	0.2	0.2	0.2	0.2	0.2
CMC	2	Overlap threshold(w)	0.7	0.7	0.7	0.7	0.7	0.7	0.7	0.7
		Merge threshold(m)	0.5	0.5	0.4	0.3	0.5	0.5	0.4	0.5
		Minimum degree ratio(c)	1	1	1	1	1	1	1	1
		Minimum size of clusters(s)	3	3	3	3	3	3	3	3
MCL	14–137	Inflation(I)	4.6	4.7	2.0	2.6	4.9	3.2	2.3	2.3
ClusterONE	1.1	Default	—	—	—	—	—	—	—	—
RNSC	Unknown(20 Apr 2010)	Shuffling diversification length(d)	9	9	3	9	5	9	9	9
		Diversification frequency(D)	50	10	20	50	50	20	20	20
		Number of experiments(e)	3	3	3	10	3	3	3	3
		Naive stopping tolerance(n)	50	20	50	50	10	20	10	20
		Scaled stopping tolerance(N)	5	15	5	1	5	5	5	1
		Tabu length(t)	100	100	50	50	100	100	10	50
		Tabu tolerance(T)	1	1	1	1	1	5	3	1
RRW	Unknown(1 Sep 2014)	Restart probability(r)	0.5	0.6	0.5	0.5	0.5	0.6	0.5	0.5
		Overlap threshold(overlap)	0.2	0.1	0.2	0.2	0.2	0.1	0.2	0.2
		Early cutoff(lambda)	0.5	0.6	0.6	0.7	0.5	0.6	0.6	0.7
		Minimum cluster size(min)	3	3	3	3	3	3	3	3
		Maximum cluster size(max)	10 000	10 000	10 000	10 000	10 000	10 000	10 000	10 000
IMHRC	1.0	Minimum density of clusters(min-density)	0.3	0.3	0.3	0.3	0.3	0.3	0.3	0.3
		Minimum size of cluster(min-size)	3	3	3	3	3	3	3	3
		Maximum size of cluster(max-size)	10 000	10 000	10 000	10 000	10 000	10 000	10 000	10 000
		Hub retrieving threshold(black-list)(γ)	0.036	0.011	0.001	0.008	0.001	0.008	0.001	0.0
		Hub removing threshold (black-list)(β)	0.098	0.042	0.004	0.015	0.004	0.01	0.002	0.001
		Overlap threshold(max-overlap)	0.59	0.88	0.82	0.81	0.53	0.86	0.80	0.81
		Growing penalty(growth-penalty)	2	2.2	3.3	2.3	2.1	4.1	2.6	2.1
		Hub retrieving penalty(back-penalty)	3.6	0.8	2	2	0.1	2.5	2.1	2

**Table 4:** Experimental results and performance comparison of all methods used in this paper on the SGD gold standard.

Gold Standards	Dataset	Algorithm	#complexes	#clusters	#matches	Minimum Size of Complexes	Maximum Size of Complexes	Minimum Size of clusters	Maximum Size of clusters	Fraction	sensitivity	PPV	Acc	MMR	Total
SGD	Collins	AP	137	202	93	3	32	3	27	**0.839**	0.783	0.660	0.719	0.502	2.060
		Cfinder		114	68			3	358	0.613	**0.858**	0.490	0.648	0.396	1.657
		CMC		327	94			3	68	0.810	0.770	0.612	0.687	**0.522**	2.019
		MCL		181	90			3	78	**0.839**	0.799	0.654	0.723	0.494	2.056
		ClusterONE		208	91			3	111	0.810	0.847	0.635	**0.734**	0.507	2.051
		RNSC		163	91			3	69	0.810	0.758	0.667	0.711	0.506	2.027
		RRW		186	89			3	25	0.766	0.690	**0.675**	0.682	0.487	1.935
		IMHRC		193	**95**			3	45	**0.839**	0.814	0.663	**0.734**	0.511	**2.096**
	Gavin	AP	130	305	89	3	29	3	26	0.808	0.694	0.659	0.676	0.423	1.907
		Cfinder		137	67			4	93	0.615	**0.817**	0.543	0.666	0.356	1.637
		CMC		856	98			3	24	0.800	0.675	0.586	0.629	0.515	1.944
		MCL		253	85			3	36	0.754	0.698	0.676	0.687	0.431	1.871
		ClusterONE		240	89			3	29	0.792	0.792	0.623	**0.702**	0.465	1.960
		RNSC		224	77			3	35	0.738	0.738	0.660	0.698	0.429	1.865
		RRW		235	84			3	34	0.754	0.672	**0.693**	0.682	0.466	1.902
		IMHRC		259	**100**			3	23	**0.815**	0.784	0.584	0.677	**0.523**	**2.017**
	Krogan Core	AP	167	354	93	3	41	3	24	0.593	0.531	0.600	0.564	0.337	1.494
		Cfinder		115	57			3	667	0.413	0.679	0.357	0.493	0.230	1.136
		CMC		853	101			3	22	0.659	0.588	0.610	0.599	0.402	1.659
		MCL		367	96			3	49	0.641	0.687	0.590	0.637	0.344	1.621
		ClusterONE		600	110			3	54	0.683	**0.733**	0.618	**0.673**	0.399	1.755
		RNSC		261	92			3	22	0.647	0.561	0.638	0.598	0.370	1.615
		RRW		261	91			3	15	0.605	0.488	**0.671**	0.572	0.360	1.537
		IMHRC		680	**119**			3	30	**0.725**	0.723	0.595	0.656	**0.451**	**1.832**
	Krogan Extended	AP	189	377	96	3	49	3	25	0.545	0.491	0.587	0.537	0.293	1.375
		Cfinder		88	45			4	425	0.265	0.551	0.401	0.470	0.145	0.879
		CMC		2461	111			3	24	0.619	0.575	0.569	0.572	0.364	1.555
		MCL		517	91			3	47	0.492	0.585	0.604	0.594	0.244	1.330
		ClusterONE		972	111			3	81	0.577	**0.670**	0.632	**0.651**	0.323	1.550
		RNSC		305	83			3	23	0.508	0.511	0.633	0.569	0.286	1.362
		RRW		231	92			3	15	0.534	0.439	**0.656**	0.536	0.312	1.383
		IMHRC		1060	**122**			3	33	**0.640**	0.654	0.623	0.638	**0.392**	**1.671**

The bold values show the best results in comparison with other methods.

**Table 5:** Experimental results and performance comparison of all methods used in this paper on the MIPS gold standard.

Gold Standards	Dataset	Algorithm	#complexes	#clusters	#matches	Minimum Size of Complexes	Maximum Size of Complexes	Minimum Size of clusters	Maximum Size of clusters	Fraction	sensitivity	PPV	Acc	MMR	Total
MIPS	Collins	AP	127	196	72	3	74	3	29	0.756	0.537	0.475	0.505	0.341	1.602
		Cfinder		114	54			3	358	0.575	**0.679**	0.360	0.494	0.281	1.350
		CMC		327	71			3	68	0.732	0.560	**0.494**	0.526	0.347	1.605
		MCL		183	73			3	71	0.740	0.598	0.465	0.527	0.353	1.620
		ClusterONE		208	73			3	111	**0.787**	0.660	0.461	**0.552**	**0.355**	**1.694**
		RNSC		160	67			3	71	0.740	0.564	0.463	0.511	0.330	1.581
		RRW		186	66			3	25	0.724	0.487	0.464	0.475	0.333	1.533
		IMHRC		188	**74**			3	73	0.764	0.647	0.462	0.547	**0.355**	1.688
	Gavin	AP	122	305	66	3	77	3	26	0.705	0.460	**0.473**	0.466	0.305	1.476
		Cfinder		137	53			4	93	0.582	**0.599**	0.397	0.488	0.251	1.320
		CMC		856	71			3	24	**0.770**	0.455	0.451	0.453	0.332	1.555
		MCL		253	66			3	40	0.705	0.520	0.472	**0.495**	0.298	1.499
		ClusterONE		240	68			3	29	0.730	0.541	0.445	0.491	0.332	1.552
		RNSC		232	61			3	34	0.648	0.501	0.461	0.481	0.299	1.427
		RRW		235	62			3	34	0.705	0.470	0.472	0.471	0.318	1.494
		IMHRC		293	**73**			3	30	0.754	0.537	0.436	0.484	**0.360**	**1.604**
	Krogan Core	AP	143	354	61	3	70	3	24	0.538	0.372	0.396	0.384	0.229	1.152
		Cfinder		115	36			3	667	0.357	**0.542**	0.241	0.361	0.142	0.860
		CMC		853	63			3	22	0.559	0.403	0.392	0.397	0.238	1.195
		MCL		376	68			3	39	0.601	0.463	0.409	0.435	0.247	1.283
		ClusterONE		600	76			3	54	**0.657**	0.516	0.385	0.446	0.272	1.375
		RNSC		204	53			3	21	0.503	0.365	**0.427**	0.395	0.209	1.108
		RRW		261	59			3	15	0.503	0.315	0.424	0.365	0.216	1.085
		IMHRC		628	**80**			3	30	0.650	0.507	0.404	**0.453**	**0.302**	**1.456**
	Krogan Extended	AP	162	321	60	3	78	3	23	0.432	0.329	0.392	0.359	0.190	0.982
		Cfinder		121	27			3	1312	0.216	**0.624**	0.155	0.311	0.095	0.622
		CMC		2565	72			3	22	0.537	0.372	0.369	0.370	0.226	1.133
		MCL		483	63			3	60	0.438	0.421	0.393	0.406	0.180	1.025
		ClusterONE		972	79			3	81	0.525	0.471	0.374	0.420	0.235	1.180
		RNSC		284	60			3	22	0.488	0.349	0.421	0.383	0.200	1.070
		RRW		231	60			3	15	0.475	0.297	**0.424**	0.355	0.194	1.024
		IMHRC		1041	**90**			3	34	**0.556**	0.453	0.395	**0.423**	**0.290**	**1.289**

The bold values show the best results in comparison with other methods.

Similarly, the number of predicted complexes often were increased when methods were implemented on sparser datasets. This process was more evident about CMC, IMHRC, Cluster ONE and MCL respectively. However, this was not true about CFinder. In contrast, it did not seem any specific patterns for increasing the number of matched clusters from denser datasets to sparser datasets, except in Cluster ONE and IMHRC. In fact, Cluster ONE and IMHRC were the only two methods whose matched predicted clusters increased when the number of predicted clusters increased. In addition, the number of matched predicted clusters which were introduced by IMHRC was always more than other methods. The Fraction criterion clearly shows which methods are more powerful in recognizing real complexes. Based on Tables 4 and 5, IMHRC, Cluster ONE and CMC have the first, second and third best performance on the Fraction criterion respectively.

**Figure 3:**
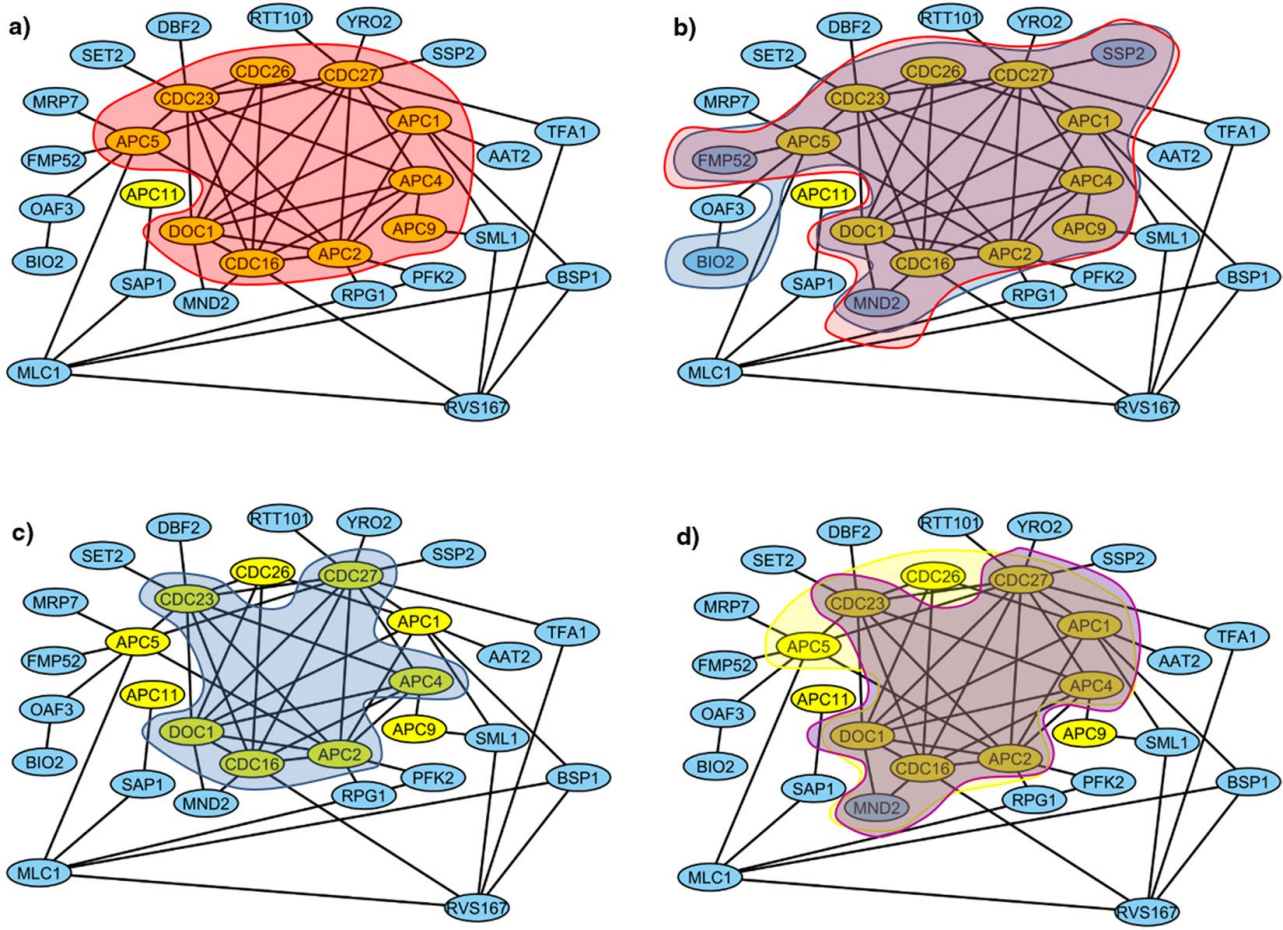
In this figure, we show results of all clustering methods on detection of a real complex based on the Krogan Extended dataset. The yellow nodes denote real complex and the blue nodes are others proteins. In addition, the halos represent results of algorithms. (**a**) The red halo shows the result of IMHRC. (**b**) The red and blue halos show the result of MCL and ClusterONE respectively. (**c**) The blue halo shows the results of RRW, RNSC and CMC. (**d**) The yellow and violet halos show the result of CFinder and AP respectively.

**Figure 4:**
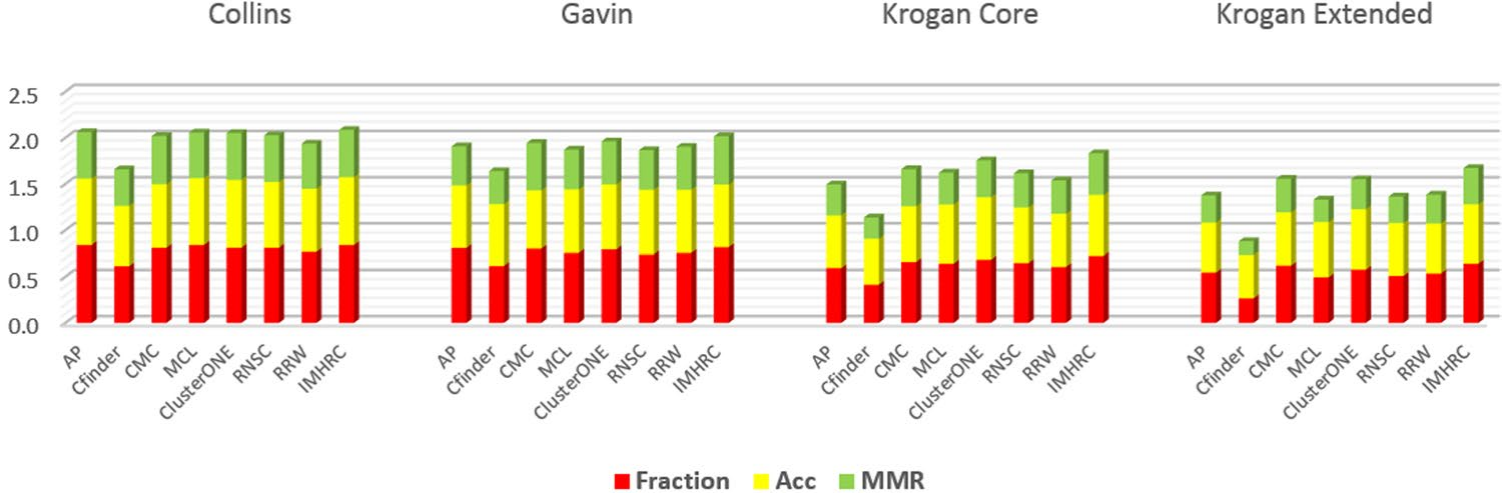
Modules obtained by different methods. Comparison of the total performance of all methods used in the evaluation on all datasets and using the SGD gold standard.

**Figure 5:**
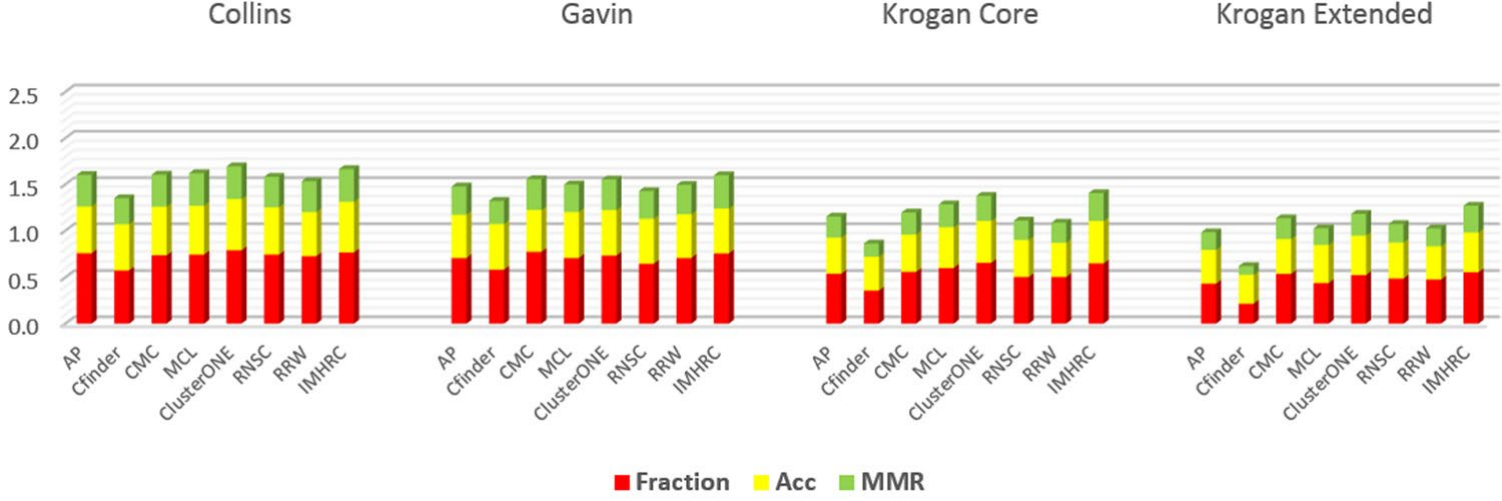
Modules obtained by different methods. Comparison of the total performance of all methods used in the evaluation on all datasets and using the MIPS gold standard.

It is notable that the number of the matched clusters in Tables 4 and 5 is the cardinality of a maximal one-to-one matching between real complexes and predicted clusters based on MMR criterion. Fraction calculates how many real complexes are recognized by at least one of the predicted clusters of a method. So considering the quantity of matched clusters is stricter than the value of Fraction criterion. Size and quality of predicted clusters are other important issues that were measured by Acc and MMR. It is obvious that a predicted cluster is more valuable if the number of its common proteins with the proteins of real complexes is high. The Sn criterion calculates the amount of matching. As it is evident in Tables 4 and 5, CFinder, ClusterONE, IMHRC, and MCL have the highest Sn value. But we know that if a method produces a giant component between its predicted clusters, the value of Sn is not completely trustable. The results in Tables 4 and 5 show CFinder and somewhat ClusterONE have such a behavior. As mentioned previously, using PPV criterion is a way for resolving this defect. A significant difference between the value of Sn and PPV for CFinder is a proof of this claim. Hence, we had to use Acc for comparing the performance of methods. The results showed that ClusterONE, IMHRC, and MCL are the first, second and third best algorithms in the terms of Acc respectively. MMR was the last criterion for comparing the performance of methods. This criterion clearly indicated how much a method could detect real complexes based on both quality and quantity. Again Tables 4 and 5 clearly represent IMHRC, CMC, and ClusterONE as the first, second and third best methods in the terms of MMR criterion. So these algorithms have more accuracy to distinguish and fit predicted clusters with real complexes. For example, we investigated one of the real complexes in the MIPS, based on the Krogan Extended dataset whose proteins are: APC5, CDC23, CDC26, CDC27, APC1, APC4, APC9, APC2, CDC16, DOC1 and APC11. The matching score 0.909, 0.736, 0.699, 0.649, 0.556, 0.545, 0.545 and 0.545 was achieved by IMHRC, CFinder, MCL, ClusterONE, AP, RRW, RNSC and CMC algorithms respectively. Figure 3 depicts the clusters obtained by these algorithms which are matched with the real complex. Finally as Figs 4 and 5 demonstrate, IMHRC dominates all other methods on all datasets except in one case. Actually, the performance of IMHRC is lower than clusterONE, when MIPS and Collins are gold standard and dataset respectively.

## Discussion

Protein complexes are fundamental operating units in cells. Therefore, understanding the characteristics and behavior of cells depend on analyzing proteins and their complexes. Many computational methods have been proposed to detect protein complexes from PPI networks.

In this paper, we propose a new complex detection algorithm which recognizes real complexes from PPI networks by removing inter-module hubs. Removing hubs are one of the fundamental parts of this algorithm. In fact, we observed that the existing noise in PPI networks and different time of protein interactions are two basic challenges for detecting real complexes from PPI networks. Our survey show removing and putting some part of hubs back can be a good alternative for overcoming these two problems. Actually, module hubs are fundamental units in the structure of complexes and perform many tasks such as “RNA metabolic process”, or “nuclear organization and biogenesis”. Therefore, the presence of them in the complexes is required, but the roles of inter-module hubs are less important in the duty of complexes. In other words, inter-module hubs have mediator roles such as “signal transduction”^14^.Our study show that many of the high degree nodes in dense PPI networks are inter-module hubs while these nodes are module hubs in sparse PPI networks. This is in agreement with the researches on hubs by Han *et al*.^27^ and the results of Liu *et al*. on the DIP dataset which is really a sparse network^14^.

We also created a powerful mechanism which is capable of considering the weights of protein interactions and overcoming the overlap property of complexes. For assessing the effect of removing hubs and robustness of our mechanism, we performed detailed evaluation. We compared our method with seven state-of-the-art techniques on four popular datasets. The results showed that not only did our method not only have the highest number of matches in all cases, but also the quality of these matches were better than the other methods. Therefore, our method can predict real complexes with more accuracy and precision.

The evaluation in our comparison was based on three common criteria which have been used in literature. But it seems that there are still some defects in these criteria that prevent us from a flawless assessment. The effect of a giant component and the number of predicted complexes are samples of imperfections. Therefore, one of our future works will be designing a mixture of criteria with fewer defects. In addition, we will try to redesign our complex detection mechanisms for detecting real complexes with more accuracy and precision.

### Application

For accessing a rigorous analysis, we performed an assessment for other algorithms the same as IMHRC algorithm. For this purpose, we removed *β* percent of vertices of network according to step 1 of IMHRC Algorithm section. Next, we run all algorithms on new network. After that, we put *γ* percent of eliminated hubs back according to the repairing phase of step 3 of IMHRC Algorithm section. Because the calculations were too long, we run them upon one gold standard – SGD – and two datasets that one is denser – Collins – and the other one is sparser – Krogan Core -. So we were able of understanding how removing and putting hubs back affect the performance. The results are depicted in Figs 1 and 2 and Supplementary Figures 1 to 20.

As shown by Figs 1 and 2 and Supplementary Figures 1 to 20, this idea can improve performance of all the algorithms except CFinder on the Collins dataset. We can partition results into three groups. The first group includes the algorithms that have significant improvement. The algorithms in the second group, have satisfactory improvement and all algorithms with partial improvement are placed in the third group. According to this classification, we placed IMHRC, ClusterONE, RNSC on the Collins dataset and MCL, CFinder on the Krogan Core, in the first group when the SGD was used as gold standard. The second group included IMHRC on the Gavin dataset and AP, CMC, RRW on the Collins dataset and also AP, RNSC, CMC, RRW on the Krogan Core dataset when the SGD was used as gold standard. Finally, all remained cases were placed in the third part. The results of IMHRC show that the improvement is partial when MIPS is the gold standard. Analyzing the results based on SGD gold standard shows that, MCL, CMC and RRW on the Collins Dataset have the best performance when we remove a lot of hubs and don’t put them back. Whereas, MCL, CFinder and RRW on the Krogan Core dataset have the best performance when we removed a lot of hubs and put them back. Nevertheless, IMHRC, ClusterONE and RNSC on the Collins dataset have the best performance when almost half of the eliminated hubs would be put back. In some cases, we need to eliminate a few hubs for accessing the best performance. For example, AP and RNSC on the Krogan Core dataset are two cases to name. For these cases, we also need to put eliminated hubs back. But it didn’t need to do for IMHRC on the Gavin, AP on the Collins, and CMC on the Krogan Core. Table 6 shows the performances of all algorithms after removing and putting hubs back.

**Table 6:** Influence of removing and putting hubs back in all methods used in this paper.

Dataset	Algorithm	Old performance	New performance	Difference of performances	β	$\gamma$
Collins	AP	2.060	2.069	+0.009	0.044	0.014
	CFinder	1.657	1.657	0	Affectless	Affectless
	CMC	2.019	2.027	+0.008	0.116	0.006
	MCL	2.056	2.058	+0.002	0.18	0.046
	ClusterONE	2.051	2.104	+0.053	0.098	0.036
	RNSC	2.027	2.098	+0.071	0.09	0.02
	RRW	1.935	1.947	+0.012	0.104	0.01
Krogan Core	AP	1.494	1.698	+0.204	0.05	0.034
	CFinder	1.136	1.208	+0.072	0.12	0.104
	CMC	1.659	1.678	+0.019	0.012	0.006
	MCL	1.621	1.699	+0.078	0.118	0.106
	ClusterONE	1.755	1.774	+0.019	0.005	0.001
	RNSC	1.615	1.643	+0.028	0.006	0.0
	RRW	1.537	1.554	+0.017	0.094	0.008

## Methods

### Terminologies

As mentioned, mathematically, a PPI network is modeled as an undirected weighted graph $G=(V,E,W)$ where *V* is a set of nodes, $E=\{{e}_{ij}:i,j\in V\}$ is a set of edges and $W:E\to { {\mathcal R} }^{+}$ is a function that assigns a weight (a positive value between 0 and 1) to every edge in the graph, in a way that nodes denote the proteins, edges denote interactions between proteins and the weights denote credibility of interactions. In this model, every ${C}_{k}=({V}_{k},{E}_{k},{W}_{k})$ where ${V}_{k}\subseteq V$, ${E}_{k}\subseteq E$, ${W}_{k}\subseteq W$ shows *k*_*th*_ cluster or subgraph which is distinguished by a graph clustering algorithm. For any protein $\,v$ $\in V$, $N(v)=\{a|va\in E\}$ is a set of neighbors of $v$ and $deg(v)=|N(v)|$ is the degree of $v$. Let $w\,(i,j)$ indicates the weight of *e*_*ij*_ and $A=[{a}_{ij}]$ indicate adjacency weighted matrix of $G$ which is defined by:

6 $${a}_{ij}=\{\begin{matrix}w(i,j),if\,(i,j)\in E\\ 0,\,otherwise\end{matrix}$$

Also, the weighted degree of node *i* is defined by as:

7 $$de{g}_{w}(i)=\sum _{j\in N(i)}{a}_{ij}$$

We defined the weighted degree of a predicted cluster *C*_*k*_ as:

8 $$de{g}_{w}({C}_{k})=de{g}_{w}^{in}({C}_{k})+de{g}_{w}^{out}({C}_{k})$$

In which $de{g}_{w}^{in}({C}_{k})$ and $de{g}_{w}^{out}({C}_{k})$ are inner weighted degree and outer weighted degree of cluster *C*_*k*_ respectively and defined as follows:

9 $$de{g}_{w}^{in}({C}_{k})=\frac{1}{2}\sum _{i\in {V}_{k}}\sum _{j\in N(i){\cap }^{}{V}_{k}}{a}_{ij}$$

10 $$de{g}_{w}^{out}({C}_{k})=\sum _{i\in {V}_{k}}\sum _{j\in N(i)-{V}_{k}}{a}_{ij}$$

Also the density of *C*_*k*_ is defined by ref. 36:

11 $$de{n}_{w}({C}_{k})=\frac{2\times de{g}_{w}^{in}({C}_{k})}{(|{V}_{k}|)(|{V}_{k}|-1)}$$

In addition, quantifying the extent of overlap between two clusters A and B were calculated in accordance with neighborhood affinity score that is defined as^3^:

12 $$O\,(A,B)=\frac{{|A{\cap }^{}B|}^{2}}{|A|\times |B|}$$

### IMHRC Algorithm

In our approach, we detected complexes in four steps. These steps were designed to reduce noises in the network, process weighted graphs, consider the property of the overlapped complexes and indirectly consider times of interaction occurrence.

**Figure 6:**
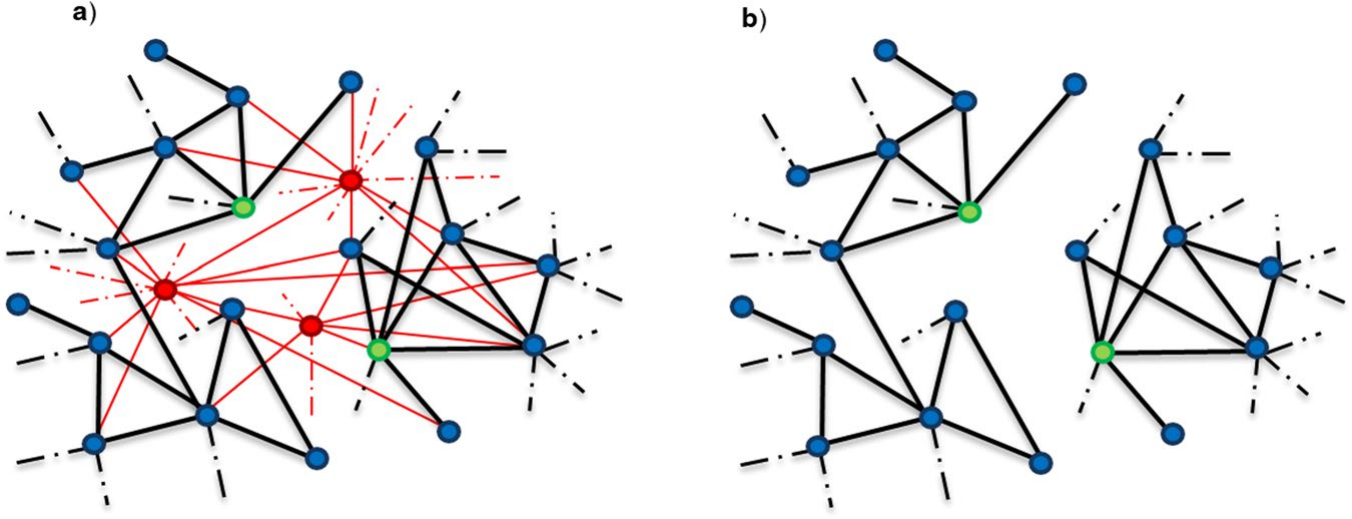
Removing hubs decreases noise and make the graph sparser. The red nodes in the figure (**a**) denote inter-module hubs and the green nodes denote module hubs in the network. When we remove inter-module hubs from the network, we will eliminate some noise and will have sparser and well-separated graph (**b**).

**Figure 7:**
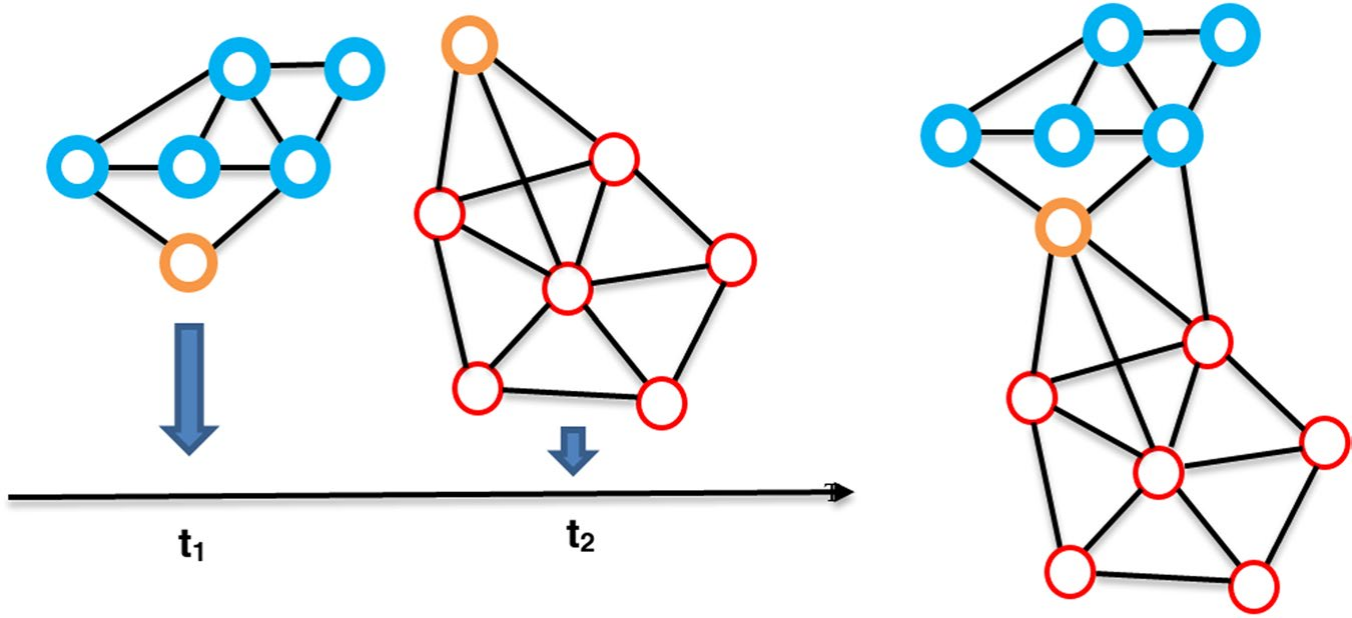
Effect of eliminating occurrence time of different interactions. The probability of creating wrong dense subgraph and hub vertices are increased when the time is not considered. The orange node is a sample that shows this situation. This normal node is a member of two dense complexes while it changes to a hub and a member of a larger subgraph in PPI network.

Step 1: In this step *β* percentage of the vertices with the highest degree (hub) were removed from the PPI network. The intuition behind the hub removal was based on two effects. First, since the occurrence probability of interactions are independent and identically distributed (i.i.d), the higher a node’s degree is, the more likely to have false positive interactions. Therefore, removing the nodes with a high degree (hub) can eliminate much more false positive interactions (Fig. 6). Second effect is to add the time asynchronization to the network implicitly. Since biological interactions occur in different times^14^, many interactions may have different times. But the weighted graph which is constructed from PPI network is static and it can’t distinguish the time of different interactions^31^. In this situation, it is possible that a vertex with low or normal degree which is common between two or more complexes turns into a vertex with a high degree or hub which is known as an inter-module hub. In fact, it is possible for a dense subgraph to rises from integrating a number of smaller and sparser subgraphs, with common vertex (Fig. 7)^28^. Our study show that such a dense subgraph is recognized wrongly as a big complex, by many methods. Although we know that this isn’t a comprehensive rule for all hubs (such as module hubs) in the main graph, a significant amount of hubs in the network behaves as mentioned. In other words, there is not a specific threshold that separates module hubs and inter-module hubs. By removing hubs, we expected that not only have the effects of ignored occurrence time of biological interactions been reduced, but also the new manipulated graph has less noise and is less complicated. In this way, we have a sparser graph of which the dense subgraphs are more obvious and complexes can be more easily detected. To implement this step, we constructed a new graph by removing hubs. In order to select hubs to be eliminated, we sorted them in the priority queue based on their degree and select *β* percentage of them from the head of the queue.

Step 2: In this step we tried to recognize primary clusters. In many methods it is a common assumption that complexes are considered as dense subgraphs^24^. We used this idea to detect the primary clusters, too. These clusters might have overlapped properties. In our definition, density was a concept that is a composition of structure and weights of edges. On the other hand, a dense subgraph not only is well separated from the rest of the network, but also its inner edges have more weight than outer edges. For detecting these subgraphs, we used a quality function named “modularity function”. The main idea in this step was based on maximizing modularity function in the form of local and greedy. The Value of modularity function for subgraph *C*_*k*_ was calculated by the equation (13).

13 $$Q({C}_{k})=\frac{de{g}_{w}^{in}({C}_{k})}{de{g}_{w}^{in}({C}_{k})+de{g}_{w}^{out}({C}_{k})+p|{C}_{k}|}$$

In this formula, “*p*” is a controlled variable, by which, we could model uncertainty. Actually, because of limitation in the experimental methods, all interactions have not been discovered. So “*p*” can be observed as an agent for these undiscovered interactions and implicitly consider them in the calculation of function. On the other hand, “*p*” is also a way to consider noise. In fact, this variable helped us control the sensitivity of density variation that stems from adding or removing 1 node to the subgraph. If there is significant amount of noise in the network, the density altered dramatically when 1 node is added to or removed from the spars subgraph. So, the smaller the size of subgraph is, the more effective the role of “*p*” is in adding a node to or removing a node from the subgraph. For implementing this approach, we acted as ClusterONE algorithm^13^. At first, we selected one node which was not in any available cluster and has the highest degree. This node which is called the “seed”, can create a new cluster. Next we tried to maximize the value of modularity function of the new cluster by an iterative and greedy approach. In this process, the best decision was made for maximizing the modularity function in every step. The best decision could be adding an external boundary node to or deleting an internal boundary node from current cluster. In this definition every external node which is the neighbor with at least one of the cluster nodes is called “external boundary node” of that cluster; and every internal node of a cluster which is the neighbor with at least one of a cluster’s external boundary node is called the “internal boundary node” of that cluster. After reaching to the maximal value of modularity function for a growing cluster, it was introduced as a new primary cluster. We repeated the process for the remaining nodes. This greedy process is explained in five following steps. Let ${u}_{0}$ represents the initial seed:

1. Let ${C}_{{k}_{0}}={u}_{0}$ and the step number $t=0$
2. Calculate the value of modularity function for ${C}_{{k}_{t}}$ and set ${C}_{{k}_{t+1}}={C}_{{k}_{t}}$
3. For every external boundary node *u* of ${C}_{{k}_{t}}$ calculate the modularity function for ${C}_{k}^{\prime} ={C}_{{k}_{t}}\cup \{u\}.$ If $Q({C}_{k}^{\prime}) > Q({C}_{{k}_{t+1}})$, let ${C}_{{k}_{t+1}}={C}_{k}^{\prime}$.
4. For every internal boundary node *u* of ${C}_{{k}_{t}}$ calculate the modularity function for ${C}_{k}^{\prime\prime}={C}_{{k}_{t}}-\{u\}.$ If $Q({C}_{k}^{\prime\prime}) > Q({C}_{{k}_{t+1}})$ then let ${C}_{{k}_{t+1}}={C}_{k}^{\prime\prime}$.
5. If ${C}_{{k}_{t+1}}\ne {C}_{{k}_{t}}$, let $t=t+1$ and return to step 2. Otherwise, maximal value of modularity function for ${C}_{{k}_{t}}$ is reached. Therefore, ${C}_{{k}_{t}}$ is recognized as a new primary cluster.

It should be noted that the initial seed could be eliminated from the cluster during growth process like as others nodes. In addition, every node only had one chance to be a seed of a new cluster. So the eliminated seed could no longer be considered as a seed but it could be added to another cluster in its growth process.

Step 3: After introducing primary clusters, in this step we tried to repair clusters and merged some of them. In repairing phase, *γ* percentage of eliminated hubs were considered and checked whether adding them to the primary clusters, increased the modularity functions or not. In fact, we wanted to add module hubs to the primary clusters and filter inter-module hubs. This part of hubs were selected from initially eliminated hubs which had the lower degree than the others. If the hubs are inserted in an ascending priority queue based on their degrees, in form of $({x}_{1},{x}_{2},{x}_{3},\ldots {x}_{n})$, *γ* percentage includes $({x}_{1},\ldots {x}_{\gamma n})$ nodes. In repairing phase, an iterative process was run on all primary clusters. In this process *x*_*i*_ was added to a primary cluster. If modularity function of the new cluster increased, the change was preserved and the same process was repeated on updated cluster with *x*_*i*+1_. On the contrary, if modularity function did not increase, the change did not maintain and the same process was repeated on the primary cluster with *x*_*i*+1_. It is obvious that module hubs have more chance than inter-module hubs to be added to the primary clusters. This is due to the number of outer edges which is usually more than the number of inner edges in an inter-module hub. This is often reversed for module hubs. So adding module hubs usually could increase modularity but it was not true about inter-module hubs. It is notable that index *i* was begun from 1 to $\lfloor \gamma n\rfloor$ for every primary cluster. The threshold *γ* was chosen as a value between 0 to 10 percent. Actually, our study show that *β* − *γ* percent of eliminated hubs with higher degrees are inter-module hubs. So not only does deleting this group of hubs reduce the complexity of the network, but also in this manner a significant amount of noise in the network is reduced.

After repairing phase, the clusters which had a significant overlap with each other were merged. For implementing this process, we created a new graph called “overlap graph”. In overlap graph, every cluster is indicated as a node and the amount of overlap between two clusters is represented by a weighted edge. This edge is created if the overlap value is above the overlap threshold (max-overlap). Based on the overlap graph, every pair of nodes was sorted in a priority queue according to their overlap value if they had an edge between them. Overlap value was calculated according to the equation (12). Next in finite steps, one pair popped from the head of the queue. If the overlap value of the pair was above the overlap threshold (max-overlap), they were merged and then the queue are updated with a new cluster and the old clusters are deleted. When there aren’t any pairs for merging, the process was terminated. This process demonstrates a fundamental difference between IMHRC and ClusterOne. ClusterOne partitions primary clusters into several groups. Each cluster will be put to a group, if its overlap value with at least one of the members of that group, is above the overlap threshold −0.8 as default-. Then, ClusterOne merges members of each group without any updating phase.

Step 4. In this step, all remaining clusters that contained less than three members were discarded. This approach is common in literature. In the final part, the clusters with density below 0.3, were discarded. The value of density was calculated according to equation (11).

## Electronic supplementary material

Supplementary Information 
