# Supplementary material for: Discovering overlapped protein complexes from weighted PPI networks by removing inter-module hubs
Source: Sci Rep. 2017 Jun 12;7:3247. doi: 10.1038/s41598-017-03268-w (PMC5468366; doi:10.1038/s41598-017-03268-w)
Supplement: Supplementary file 1 — Supplementary Information [file 41598_2017_3268_MOESM1_ESM.pdf]

Discovering overlapped protein complexes from weighted PPI networks by removing inter-module hubs

A. M. A. Maddi[1,3], Ch. Eslahchi[2,3,*]

[1]Department of Electrical and computer Engineering, Isfahan University of Technology, Isfahan, 1983963113, Iran., [2]Department of Computer Sciences, Faculty of Mathematics, Shahid Beheshti University, Tehran, 1983963113, Iran., [3]School of Biological Sciences, Institute for Research in Fundamental Sciences (IPM), Tehran, 193955746, Iran.

*corresponding ch-eslahchi@sbu.ac.ir

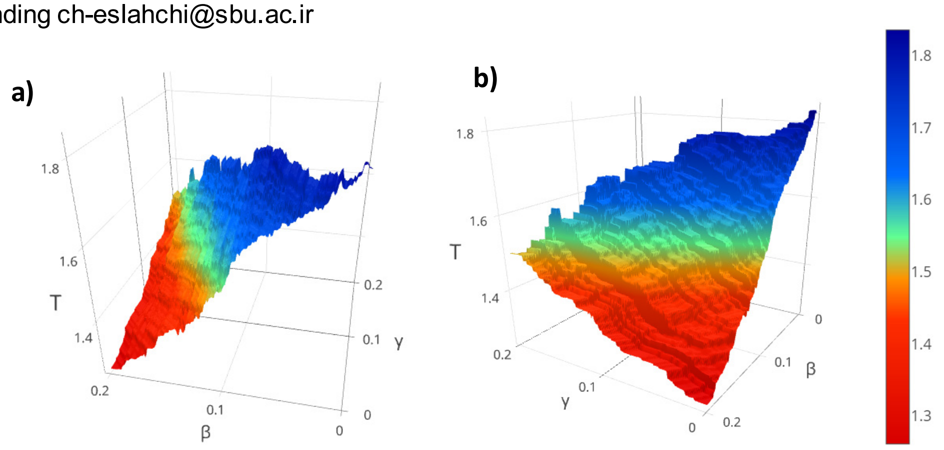

Supplementary Figure 1. Performance of IMHRC for the different values of β and γ on the Krogan Core dataset and the SGD gold standard. The β and γ axes indicate the number of hubs that have been removed and put back, respectively. The T axis specifies the performance of method. a) Side view of surface is shown in this figure. b) Front view of surface is shown in this figure.

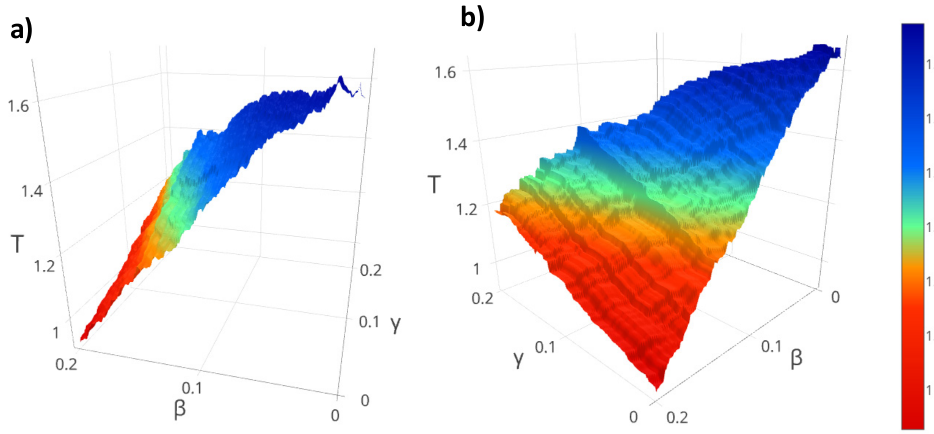

Supplementary Figure 2. Performance of IMHRC for the different values of β and γ on the Krogan Extended dataset and the SGD gold standard. The β and γ axes indicate the number of hubs that have been removed and put back, respectively. The T axis specifies the performance of method. a) Side view of surface is shown in this figure. b) Front view of surface is shown in this figure.

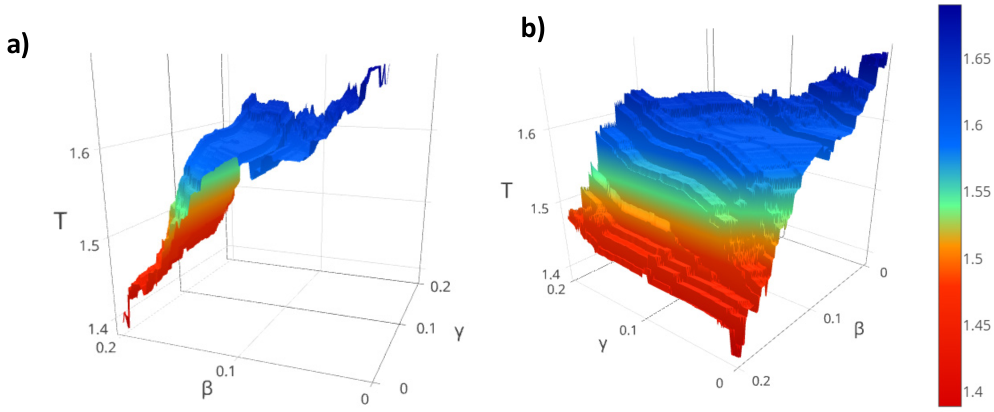

Supplementary Figure 3. Performance of IMHRC for the different values of β and γ on the Collins dataset and the MIPS gold standard. The β and γ axes indicate the number of hubs that have been removed and put back, respectively. The T axis specifies the performance of method. a) Side view of surface is shown in this figure. b) Front view of surface is shown in this figure.

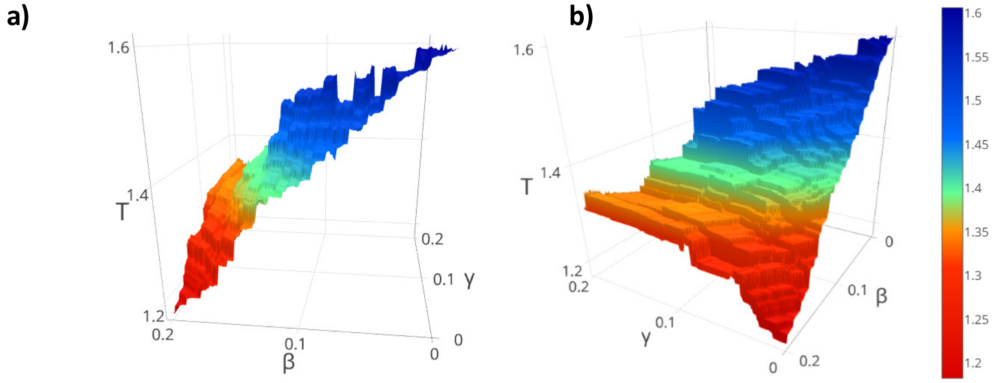

Supplementary Figure 4. Performance of IMHRC for the different values of β and γ on the Gavin dataset and the MIPS gold standard. The β and γ axes indicate the number of hubs that have been removed and put back, respectively. The T axis specifies the performance of method. a) Side view of surface is shown in this figure. b) Front view of surface is shown in this figure.

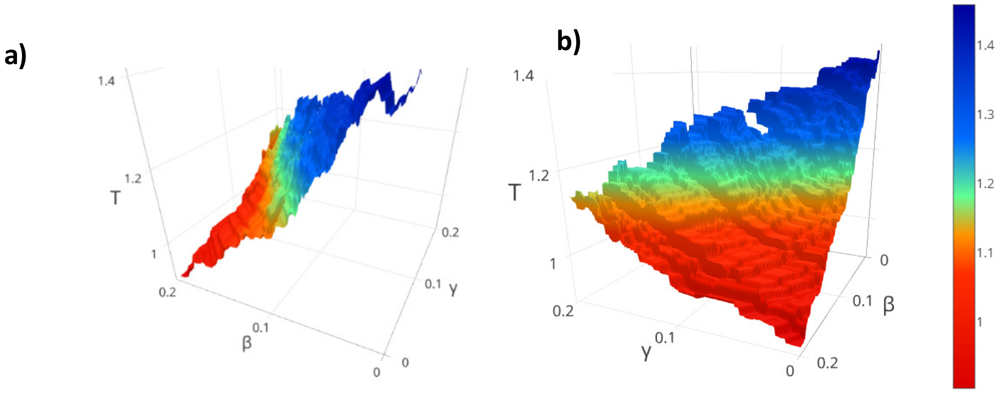

Supplementary Figure 5. Performance of IMHRC for the different values of β and γ on the Krogan Core dataset and the MIPS gold standard. The β and γ axes indicate the number of hubs that have been removed and put back, respectively. The T axis specifies the performance of method. a) Side view of surface is shown in this figure. b) Front view of surface is shown in this figure.

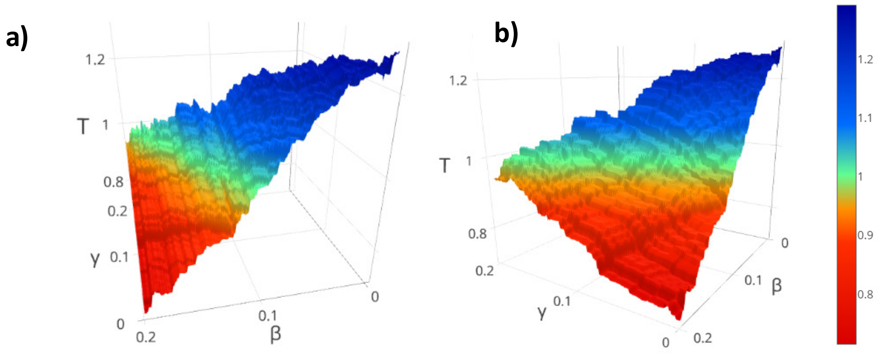

Supplementary Figure 6. Performance of IMHRC for the different values of β and γ on the Krogan Extended dataset and the MIPS gold standard. The β and γ axes indicate the number of hubs that have been removed and put back, respectively. The T axis specifies the performance of method. a) Side view of surface is shown in this figure. b) Front view of surface is shown in this figure.

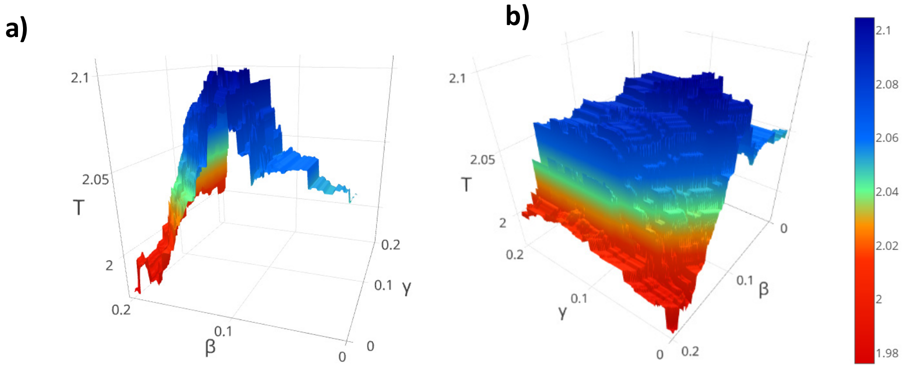

Supplementary Figure 7. Performance of ClusterONE for the different values of β and γ on the Collins dataset and the SGD gold standard. The β and γ axes indicate the number of hubs that have been removed and put back, respectively. The T axis specifies the performance of method. a) Side view of surface is shown in this figure. b) Front view of surface is shown in this figure.

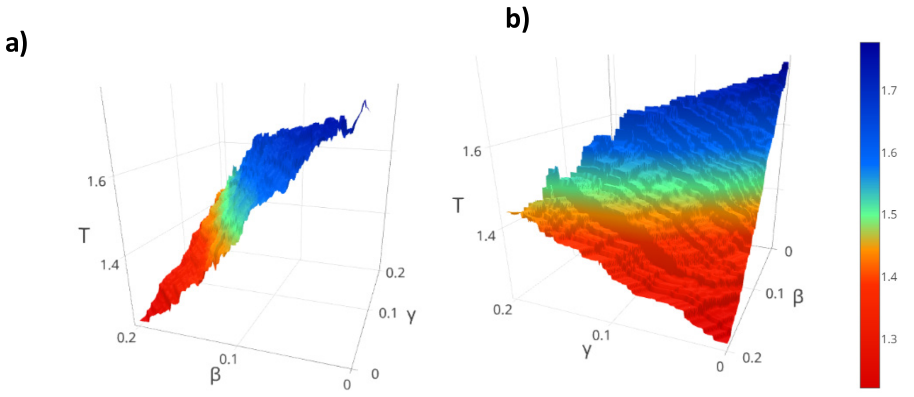

Supplementary Figure 8. Performance of ClusterONE for the different values of β and γ on the Krogan Core dataset and the SGD gold standard. The β and γ axes indicate the number of hubs that have been removed and put back, respectively. The T axis specifies the performance of method. a) Side view of surface is shown in this figure. b) Front view of surface is shown in this figure.

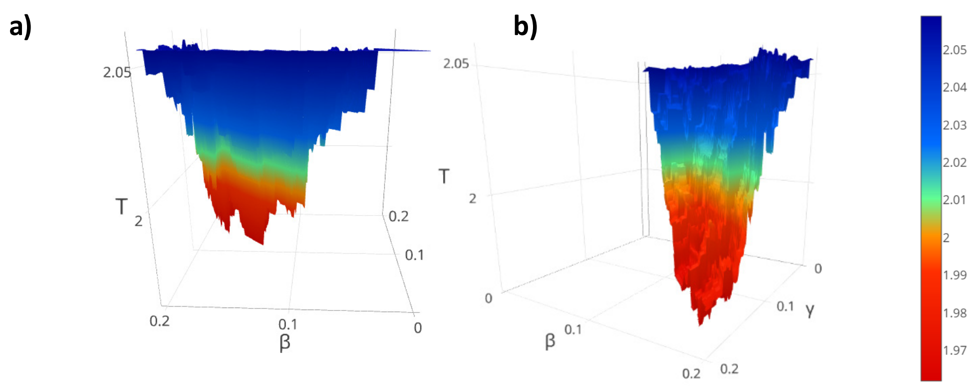

Supplementary Figure 9. Performance of MCL for the different values of β and γ on the Collins dataset and the SGD gold standard. The β and γ axes indicate the number of hubs that have been removed and put back, respectively. The T axis specifies the performance of method. a) Side view of surface is shown in this figure. b) Another side view of surface is shown in this figure.

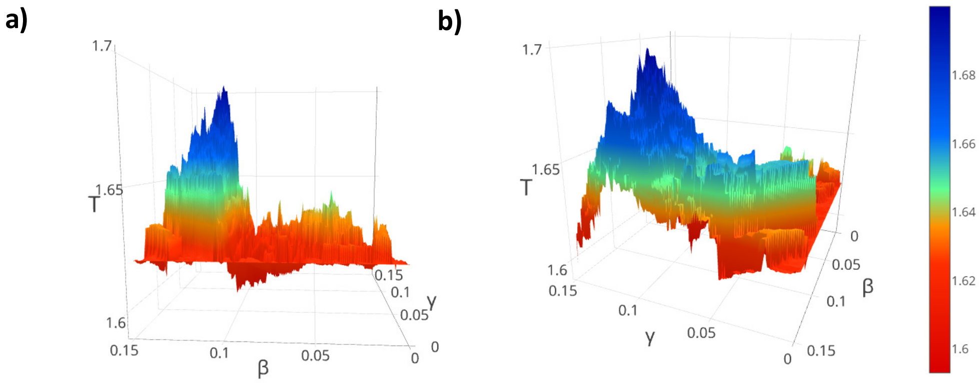

Supplementary Figure 10. Performance of MCL for the different values of β and γ on the Krogan Core dataset and the SGD gold standard. The β and γ axes indicate the number of hubs that have been removed and put back, respectively. The T axis specifies the performance of method. a) Side view of surface is shown in this figure. b) Front view of surface is shown in this figure.

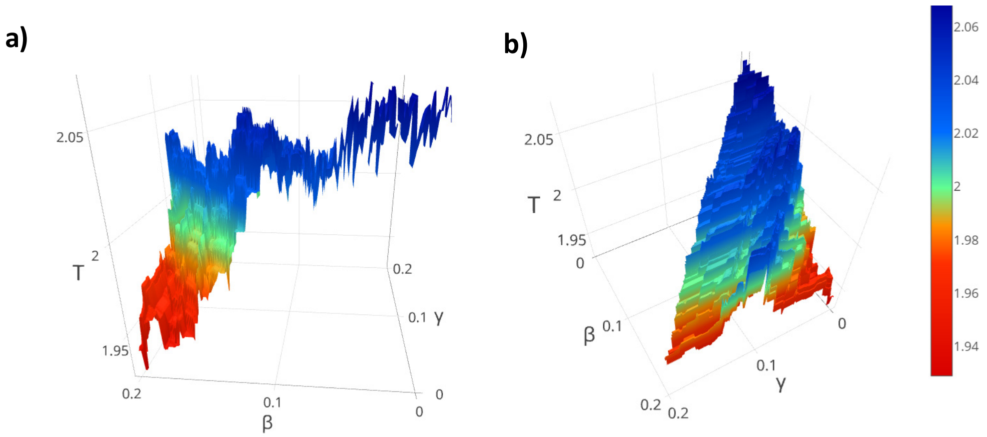

Supplementary Figure 11. Performance of AP for the different values of β and γ on the Collins dataset and the SGD gold standard. The β and γ axes indicate the number of hubs that have been removed and put back, respectively. The T axis specifies the performance of method. a) Side view of surface is shown in this figure. b) Front view of surface is shown in this figure.

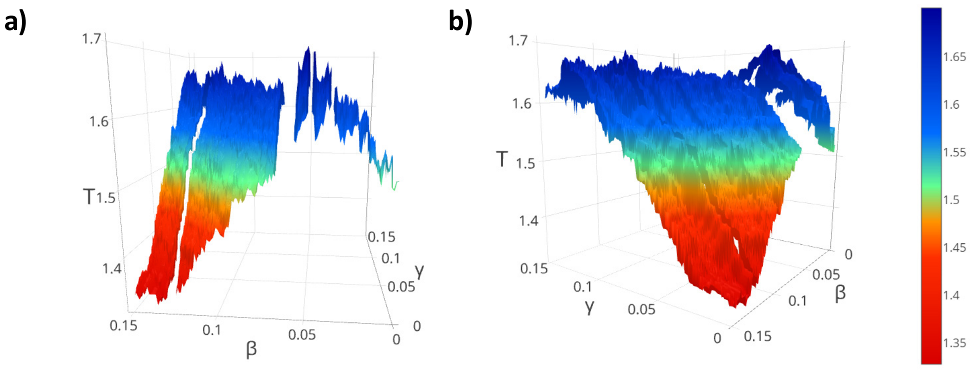

Supplementary Figure 12. Performance of AP for the different values of β and γ on the Krogan Core dataset and the SGD gold standard. The β and γ axes indicate the number of hubs that have been removed and put back, respectively. The T axis specifies the performance of method. a) Side view of surface is shown in this figure. b) Front view of surface is shown in this figure.

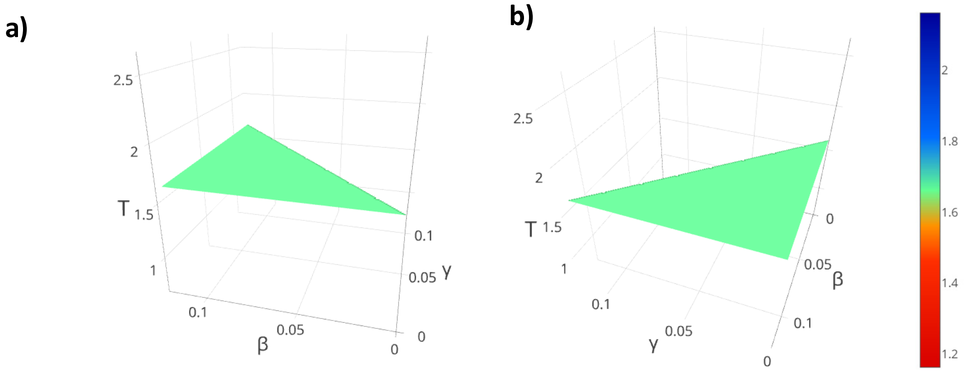

Supplementary Figure 13. Performance of CFinder for the different values of β and γ on the Collins dataset and the SGD gold standard. The β and γ axes indicate the number of hubs that have been removed and put back, respectively. The T axis specifies the performance of method. a) Side view of surface is shown in this figure. b) Front view of surface is shown in this figure.

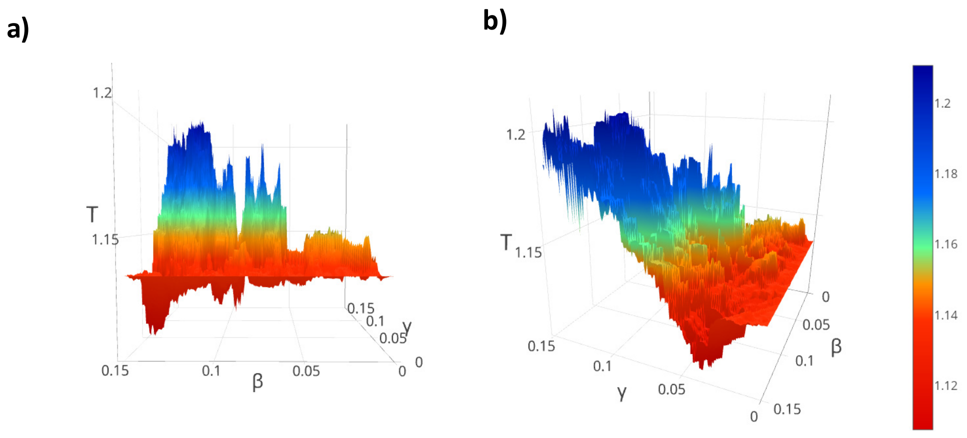

Supplementary Figure 14. Performance of CFinder for the different values of β and γ on the Krogan Core dataset and the SGD gold standard. The β and γ axes indicate the number of hubs that have been removed and put back, respectively. The T axis specifies the performance of method. a) Side view of surface is shown in this figure. b) Front view of surface is shown in this figure.

a) **b)**

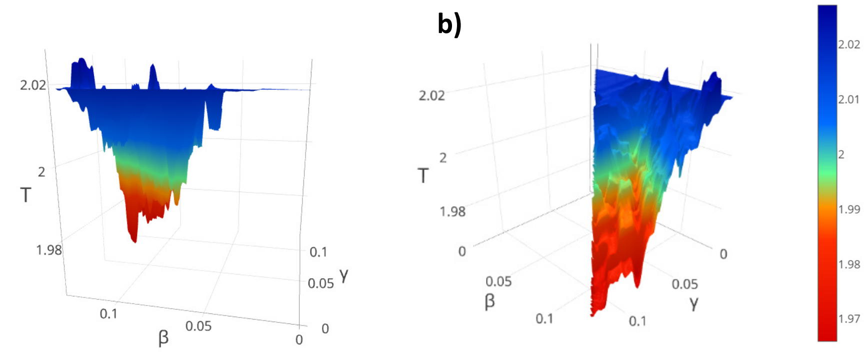

Supplementary Figure 15. Performance of CMC for the different values of β and γ on the Collins dataset and the SGD gold standard. The β and γ axes indicate the number of hubs that have been removed and put back, respectively. The T axis specifies the performance of method. a) Side view of surface is shown in this figure. b) Front view of surface is shown in this figure.

a) **b)**

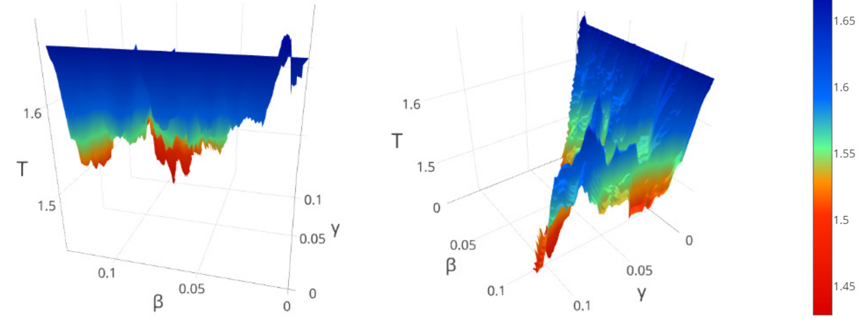

Supplementary Figure 16. Performance of CMC for the different values of β and γ on the Krogan Core dataset and the SGD gold standard. The β and γ axes indicate the number of hubs that have been removed and put back, respectively. The T axis specifies the performance of method. a) Side view of surface is shown in this figure. b) Front view of surface is shown in this figure.

a) **b)**

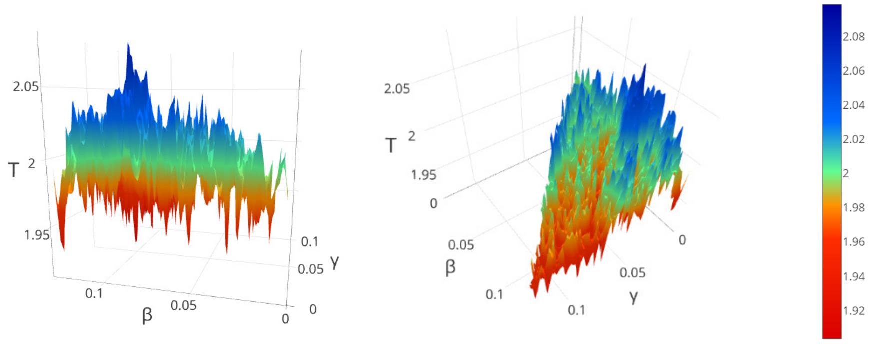

Supplementary Figure 17. Performance of RNSC for the different values of β and γ on the Collins dataset and the SGD gold standard. The β and γ axes indicate the number of hubs that have been removed and put back, respectively. The T axis specifies the performance of method. a) Side view of surface is shown in this figure. b) Front view of surface is shown in this figure.

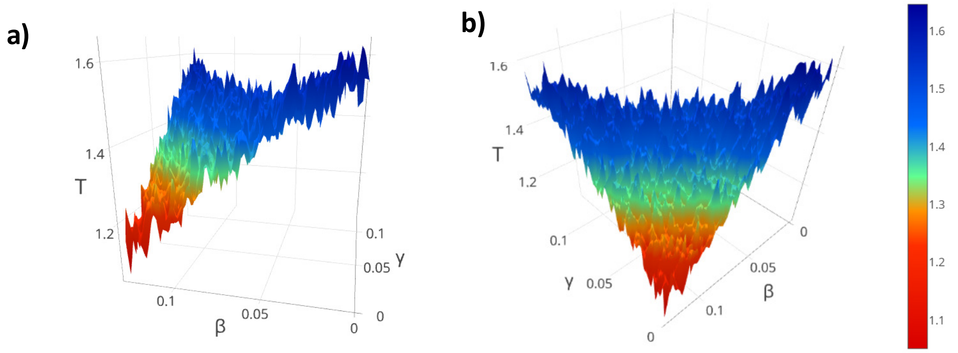

Supplementary Figure 18. Performance of RNSC for the different values of β and γ on the Krogan Core dataset and the SGD gold standard. The β and γ axes indicate the number of hubs that have been removed and put back, respectively. The T axis specifies the performance of method. a) Side view of surface is shown in this figure. b) Front view of surface is shown in this figure.

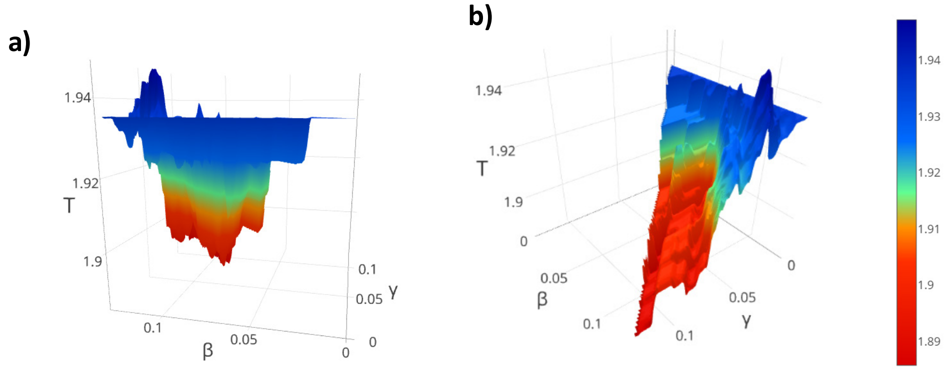

Supplementary Figure 19. Performance of RRW for the different values of β and γ on the Collins dataset and the SGD gold standard. The β and γ axes indicate the number of hubs that have been removed and put back, respectively. The T axis specifies the performance of method. a) Side view of surface is shown in this figure. b) Front view of surface is shown in this figure.

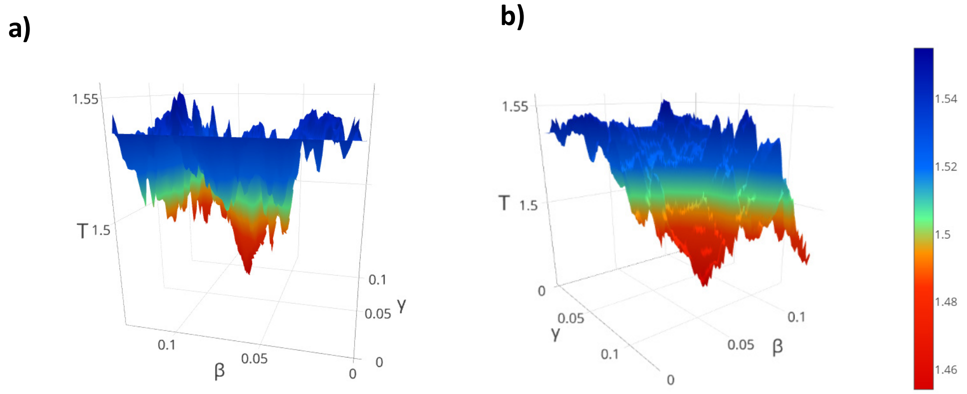

Supplementary Figure 20. Performance of RRW for the different values of β and γ on the Krogan Core dataset and the SGD gold standard. The β and γ axes indicate the number of hubs that have been removed and put back, respectively. The T axis specifies the performance of method. a) Side view of surface is shown in this figure. b) Another side view of surface is shown in this figure.
